# Prevalence of psychiatric comorbidities and treatment initiation in African American pediatric patients with vitiligo: A retrospective, single-center, case-control study

**DOI:** 10.1016/j.jdin.2024.07.012

**Published:** 2024-08-16

**Authors:** Emily Strouphauer, Sana Suhail, Carly Mulinda, Promise Ufomadu, Nicole Nyamongo, Grace Lee, Soo Jung Kim

**Affiliations:** a School of Medicine, Baylor College of Medicine, Houston, Texas; b School of Medicine, The University of Texas Health Science Center, San Antonio, Texas; c Columbia University Vagelos College of Physicians and Surgeons, New York, New York; d Departments of Dermatology and Pediatrics, Texas Children’s Hospital, Houston, Texas; e Department of Dermatology, Baylor College of Medicine, Houston, Texas

**Keywords:** pediatric dermatology, psychiatric comorbidities, psychodermatology, skin of color, vitiligo

## Abstract

**Background:**

Vitiligo may impact psychosocial development, especially among African American pediatric patients, given heightened visibility and increasing rates of anxiety and depression in this subpopulation.

**Objective:**

Evaluate psychiatric comorbidities and treatment initiation in African American pediatric patients.

**Methods:**

A total of 327 African American pediatric patients with vitiligo were each matched to 3 patients without vitiligo by age, race, and sex in this case-control study. Prevalence of psychiatric conditions and subsequent initiation of pharmacotherapy and/or psychotherapy were analyzed.

**Results:**

Compared to controls, pediatric African American patients with vitiligo were significantly more likely to be diagnosed with depression (*P* < .001) disruptive behavior disorders (*P* < .001), eating disorders (*P* = .013), generalized anxiety disorder (*P* < .001), substance abuse (*P* = .011), and suicidal ideation (*P* = .005). Patients with depression, disruptive behavior disorders, and eating disorders had higher initiation rates (76.5%, 82.1%, and 100%, respectively) for psychiatric treatment compared to those with generalized anxiety disorder and substance abuse (55.3% and 61.5%). Nearly 15% of patients did not initiate treatment for suicidal ideation.

**Limitations:**

This retrospective study has a limited sample size in a single institution and does not explore psychiatric treatment efficacy.

**Conclusions:**

Better understanding of associated psychological comorbidities and impacts on African American children of vitiligo may improve quality of life and dermatologic outcomes for these individuals.

**Capsule Summary**

• Vitiligo negatively affects quality of life, yet there has been limited investigation into psychiatric comorbidities and behavioral health treatment initiation in African American pediatric populations.
• Understanding psychiatric comorbidities in African American children with vitiligo may help dermatologists recognize pediatric mental health needs and prompt them to offer appropriate interventions and referrals.

## Introduction

Vitiligo is an acquired, chronic autoimmune disorder characterized by cutaneous depigmentation resulting from progressive destruction of pigment-producing melanocytes.^1^ Milky-white patches, characteristic of vitiligo, emerge on diverse anatomical distributions, commonly the hands, arms, elbows, knees, and shins.^2^ The condition is classified as segmental or nonsegmental vitiligo, with segmental affecting 1 area and generalized, constituting 50% to 90% of nonsegmental cases, appearing in multiple anatomical regions.^3^ The estimated global prevalence of vitiligo is 0.5% to 2.0% in pediatric populations, without sex differences.^1^^,^^4^ While some studies suggest a lack of predilection across ethnic and racial groups, a recent cross-sectional study of roughly 3 million patients with vitiligo identified varying prevalence rates among racial and ethnic minority groups: 0.29% in Asian Americans, 0.23% in Blacks, 0.29% in Hispanics/Latinos, and 0.14% in Whites.^5^^,^^6^ For pediatric patients, vitiligo often manifests early in life, with 50% of cases presenting before age 20.^7^^,^^8^

The impact of vitiligo extends beyond physical presentations, affecting the psychosocial well-being of affected children who face unique challenges during critical periods of psychological development.^9^ Social stigmatization, often arising from misconceptions of contagion or poor hygiene,^10^ contributes to increased psychosocial stressors, leading to impaired quality of life (QoL).^7^^,^^10^, ^11^, ^12^ A larger affected body surface area and visible locations of lesions on the face, neck, arms, and legs make QoL substantially worse.^4^^,^^13^ Additionally, psychosocial stressors may lead to disease flares.^13^ Importantly, the impact of adverse experiences linked to vitiligo in childhood, such as social isolation, feelings of shame, and avoidance of intimacy, can persist into adulthood.^9^

While research on vitiligo in children is limited, existing data suggest that they may experience higher rates of psychological comorbidities, particularly depression and anxiety, in comparison to healthy peers.^7^^,^^11^^,^^14^^,^^15^ Moreover, the prevalence of comorbidities observed in adults with vitiligo, such as body dysmorphic disorder, substance abuse (SA), and suicidal ideation (SI) remains inadequately characterized in children with vitiligo, which underscores the need for comprehensive mental health support in this susceptible demographic.^11^^,^^12^^,^^16^^,^^17^

While vitiligo can be distressing for patients of all skin types, due to the depigmenting nature of the disease, patients with darker skin types (Fitzpatrick skin phototypes IV-VI) report significantly higher psychosocial burden compared to their lighter-skinned counterparts.^4^^,^^10^^,^^18^ This reality poses a compounded risk for African American pediatric patients with vitiligo, as the rates of anxiety and depression in African American adolescents have doubled over the past several decades.^19^ Lifetime prevalence estimates of mood and anxiety disorders are 13.4% and 33.4%, respectively, in African American patients under the age of 18.^19^ Despite these rates, African American children receive significantly less medical treatment for psychiatric conditions than White children.^20^

Despite the evident need for an understanding of the psychosocial impact of vitiligo on African American children, there is a lack of research addressing this intersectional population. This study aims to fill this gap by assessing both the prevalence and treatment rates for psychiatric comorbidities among African American children and adolescents with vitiligo. Research in this area is crucial for developing targeted interventions to address the unique psychosocial challenges encountered by this vulnerable patient group, ultimately enhancing both their QoL and dermatologic outcomes.

## Methods

### Study design, setting, and participants

This retrospective study included pediatric, African American patients with a diagnosis of vitiligo at Texas Children Hospital in Houston, Texas between January 2012 and December 2022, forming the case cohort. Patients who self-identified as African American with a diagnosis of vitiligo were selected through SlicerDicer, a data exploration tool, in Epic electronic health record system using diagnosis code International Classification of Diagnosis, Tenth revision L80 and ninth revision 709.01. Vitiligo diagnoses were further subclassified as segmental or generalized vitiligo based on electronic health record notes documented by 1 or more board-certified dermatologists. During the chart review, we found that most patients were actively receiving first-line treatments, including topical calcineurin inhibitors and/or topical corticosteroids. Individuals under the age of 3 were excluded from the study, aligning with the common practice in pediatric psychiatry research, which typically sets the lower age limit for inclusion at 3 years.^21^ Patients aged 18 years and older were excluded, focusing on the pediatric population. To create the control cohort, each identified case participant was age-, race-, ethnicity-, and sex-matched to 3 other patients without a diagnosis of vitiligo during the same time period.

Case and control patient charts were individually screened by word search for below psychiatric diagnoses in all available patient records, which included notes from outside hospitals and clinics. The following psychiatric diagnoses were searched: bipolar disorder (I/II), body dysmorphic disorder, excoriation, trichotillomania, depression, disruptive behavior disorders (DBDs), eating disorders (EDs), generalized anxiety disorder (GAD), obsessive compulsive disorder, panic disorder, schizophrenia, SA, and SI. Diagnoses made by a psychiatrist or primary care provider after the vitiligo was diagnosed were included. Psychiatric diagnoses preceding the diagnosis of vitiligo, if applicable, were excluded.

To comprehensively assess the initiation rates of psychiatric treatment, the records of patients diagnosed with vitiligo and 1 or more psychiatric comorbidities were individually examined. The instances of subsequent psychotherapy visits and/or pharmacotherapy trial involving antidepressants, anxiolytics, antipsychotics, stimulants, and/or mood stabilizers were identified and analyzed.

### Statistical analysis

Odds ratios of each psychiatric diagnosis were calculated at 95% confidence intervals (CIs) to measure the association of each psychiatric comorbidity in African American pediatric patients with vitiligo. A multivariate logistic regression was performed to assess the relation between 1 or more psychiatric comorbidities and the explanatory variables: anatomical extent of vitiligo (categorized as either segmental or generalized) and sex. Data were checked for multicollinearity with the Belsley-Kuh-Welsch technique. Heteroskedasticity and normality of residuals were assessed respectively by the White test and the Shapiro-Wilk test. Statistical significance was set at *P* < .05, and all analyses were performed using R Statistical Software.^22^

## Results

The study included 327 pediatric African American patients diagnosed with vitiligo and 981 control patients. The patients, with an average age of 11.7 years, exhibited a female-to-male ratio of 2:1. The demographics of case and control patients are displayed in Table I. Compared to the control cohort, pediatric African American patients with vitiligo were significantly more likely to be subsequently diagnosed with comorbid depression (odds ratio [OR] 3.63, 95% CI 2.19-6.03, *P* < .001), SI (OR 2.88, 95% CI 1.38-6.03, *P* = .005), DBDs (OR 7.68, 95% CI 4.28-13.78, *P* < .001), EDs (OR 15.22, 95% CI 1.78-130.74, *P* = .013), GAD (OR 2.61, 95% CI 1.67-4.09, *P* < .001), and SA (OR 2.67, 95% CI 1.26-5.67, *P* = .011). Of note, individual diagnoses of EDs – anorexia nervosa, bulimia nervosa, and unspecified EDs – were increased, but these results did not achieve statistical significance, although EDs collectively were significantly higher among African American pediatric patients with vitiligo. Table II presents the prevalence of each psychiatric comorbidity in both case and control cohorts, along with their respective ORs.

**Table I:** Demographics of African American pediatric patients with vitiligo and corresponding controls

Characteristics	Cases	Controls
*N*	327	981
Average age (SD)	11.719 (3.90)	11.719 (3.90)
Ethnicity (%)		
Not Hispanic or Latino	300 (91.74)	900 (91.74)
Hispanic or Latino	24 (7.34)	72 (7.34)
Unable to obtain	3 (0.92)	9 (0.92)
Sex (%)		
Female	204 (62.39)	612 (62.39)
Male	123 (37.61)	369 (37.61)

*SD*, Standard deviation.

**Table II:** Psychiatric comorbidities of African American pediatric patients with vitiligo and control participants

Psychiatric comorbidity	Cases	Controls	OR (95% CI)	*P* value
Bipolar disorder (I/II)	2	8	0.749 (0.158, 3.543)	.7149
Body dysmorphic disorder	1	0	9.018 (0.367, 221.940)	.178
Body focused repetitive behaviors	1	3	1.00 (0.104, 9.647)	1
Excoriation	1	1	3.006 (0.188, 48.199)	.437
Trichotillomania	0	2	0.598 (0.029, 12.492)	.74
Depression	34	30	3.629 (2.184, 6.031)	<.001∗
Suicidal ideation	14	15	2.881 (1.375, 6.034)	.005∗
Disruptive behavior disorders	39	17	7.679 (4.280, 13.779)	<.001∗
Eating disorders	5	1	15.217 (1.771, 130.739)	.013∗
Anorexia nervosa	3	1	9.074 (0.941, 87.542)	.057
Bulimia nervosa	0	0	2.997 (0.059, 151.349)	.583
Eating disorder - not otherwise specified	2	0	15.077 (0.722, 314.871)	.08
Generalized anxiety disorder	38	47	2.613 (1.670, 4.088)	<.001∗
Obsessive-compulsive disorder	3	2	4.532 (0.754, 27.245)	.099
Panic disorder	1	2	1.502 (0.136, 16.614)	.74
Schizophrenia	2	2	3.012 (0.423, 21.471)	.271
Substance abuse	13	15	2.666 (1.255, 5.665)	.011∗

*CI*, Confidence interval; *OR*, odds ratio.

∗ Denotes significance.

In a logistic regression analysis investigating factors influencing psychiatric comorbidity, we examined the impact of sex and anatomical extent (categorized as either segmental or generalized vitiligo) among patients within the case cohort who exhibited at least 1 psychiatric comorbidity. When comparing anatomical extents, no significant difference was found between segmental and generalized changes in dermatological conditions regarding the likelihood of psychiatric comorbidity (severity = generalized; OR = 1.08, [0.59; 1.98], *P* = .7927). Similarly, there was no significant sex effect, with ORs indicating comparable risks between males and females (sex = M; OR = 1.36, [0.81; 2.29], *P* = .2416). The results of the multivariate analysis are depicted in Table III.

**Table III:** Logistic regression analysis of factors influencing psychiatric comorbidity in African American patients with vitiligo

	OR	*P* value
Intercept		
	0.33 [0.238; 0.457]	<.0001∗
Anatomical extent		
Reference: Segmental	1.08 [0.593; 1.980]	.793
Generalized		
Sex		
Reference: Female	1.36 [0.811; 2.290]	.242
Male		

*OR*, Odds ratio.

∗ Denotes significance.

The initiation of psychotherapy, (a.k.a talk therapy that encompasses a wide range of techniques used to help individuals change distressing behaviors, thoughts, and emotions), pharmacotherapy, or a combination there of, among pediatric patients with vitiligo and 1 or more psychiatric morbidities varied among specific diagnoses (Table IV). Individuals with depression, DBDs, and EDs had higher initiation rates (76.5%, 82.1%, and 100%, respectively) of any type of psychiatric treatment. In contrast, those with GAD and SA had lower rates of treatment initiation (55.3% and 61.5%, respectively). Nearly 15% of pediatric African American patients with vitiligo and reported passive or active SI did not initiate psychiatric treatment.

**Table IV:** Therapy initiation of African American pediatric patients with vitiligo and psychiatric comorbidities

Psychiatric comorbidity	Cases	Initiation of pharmacotherapy (% of cases)	Initiation of psychotherapy (% of cases)	Initiation of combination therapy (% of cases)	Total therapy initiation (% of cases)	No therapy initiated (% of cases)
Bipolar disorder (I/II)	2	0	0	2 (100)	2 (100)	0
BDD	1	0	1 (100)	0	1 (100)	0
BFRB	1	0	0	0	0	1 (100)
Depression	34	1 (2.94)	15 (44.12)	10 (29.41)	26 (76.47)	8 (23.53)
Suicidal ideation	14	1 (7.14)	7 (50)	4 (28.57)	12 (85.71)	2 (14.29)
DBD	39	4 (10.25)	21 (53.85)	7 (17.95)	32 (82.05)	7 (17.95)
Eating disorders	5	0	4 (80)	1 (20)	5 (100)	0
GAD	38	1 (2.63)	14 (36.84)	6 (15.79)	21 (55.26)	17 (44.74)
OCD	3	0	1 (33.33)	2 (66.67)	3 (100)	0
Panic disorder	1	0	1 (100)	0	1 (100)	0
Schizophrenia	2	0	0	2 (100)	2 (100)	0
Substance abuse	13	1 (7.69)	6 (46.16)	1 (7.69)	8 (61.54)	5 (38.46)

*BDD*, Body dysmorphic disorder; *BFRB*, body focused repetitive behaviors; *DBD*, disruptive behavior disorder; *GAD*, generalized anxiety disorder; *OCD*, obsessive compulsive disorder.

Further analysis was conducted on a subset of 27 case patients who commenced either psychotherapy, pharmacotherapy, or a combination of both, to estimate continued engagement with behavioral health services beyond the initial session. On average, psychotherapy spanned 5.19 sessions (SD = 6.09), while pharmacotherapy had an average duration of 10.11 months (SD = 13.25).

## Discussion

While earlier studies have highlighted the impact of vitiligo on QoL, anxiety, and depression in children and adults,^14^^,^^23^ a comprehensive examination of psychiatric comorbidities in an African American pediatric population has not been conducted. Our study showed that pediatric African American patients with vitiligo had significantly higher rates of not only depression and anxiety but other psychiatric diseases of DBDs, EDs, SA, and SI compared to those without vitiligo. This is consistent with the prior studies of depression, GAD, and SI, emerging as some of the most extensively documented associations with vitiligo in African American pediatric patients.

This study offers novel insights into the potential association between EDs and vitiligo among African American pediatric populations. Although the cause of ED cannot be determined and is likely multifactorial, poor body image and low self-esteem created by vitiligo may put pediatric patients, and especially adolescents (peak age onset of EDs), at risk. Additionally, sleep disturbances and feelings of helplessness, hopelessness, and stigmatization often reported by patients with vitiligo can compound into a decreased sense of control, predisposing to the development of EDs.^10^ Children with chronic diseases often exhibit a reduced internal health locus of control compared to healthy peers.^24^ These vulnerabilities can also contribute to the development of impulse control disorders, including EDs, during childhood—an influential period in identity and agency formation. Moreover, it is important to consider the potential link between vitiligo, EDs, and autoimmune polyglandular syndromes. Autoimmune polyglandular syndromes, which involve multiple endocrine gland failures, could provide a biological basis for the co-occurrence of these conditions.^25^ This association warrants further investigation, as understanding the autoimmune components could lead to more comprehensive treatment strategies for affected individuals.

Disruptive behavioral disorders (DBDs) and SA were more prevalent among pediatric African American patients with vitiligo, which may indicate the use of maladaptive coping mechanisms in response to the challenges posed by vitiligo. Our finding is supported by the study that children with vitiligo had higher levels of emotional dysregulation compared to their healthy counterparts.^15^ However, diagnosing DBDs and establishing their link to vitiligo should be approached with caution: DBDs in children may manifest as aggression, defiance, or withdrawal,^26^ which can be difficult to distinguish from normal patterns of childhood development. This difficulty is especially pronounced in younger children who may struggle with identifying, understanding, and communicating feelings of anxiety or other mood disorders.^15^

Existing literature has shown that factors associated with a significantly higher psychosocial burden include female sex, visible or genital lesions, age <30 years (particularly adolescents), and greater body surface area involvement. In contrast, our study demonstrates that psychiatric comorbidities are neither correlated with the extent of anatomical involvement nor sex. Rather, the presence of vitiligo has impacted patients, suggesting that patient QoL may be more influenced by concerns about the presence of vitiligo, psychosocial adjustment, and psychiatric conditions than by clinician-perceived severity as suggested in previous studies.^27^

Psychiatric treatment initiation rates observed in this study were unsatisfactory; the majority of patients did not receive both pharmacotherapy and psychotherapy, the current gold standard for several of the included conditions.^28^^,^^29^ Studies reveal that African Americans underutilize mental health treatment compared to Whites,^30^ necessitating exploration of potential reasons. Some commonly cited barriers to psychiatric treatment in this population include low socioeconomic status such as low income, insufficient insurance coverage, access to transportation and childcare, cultural mistrust of medical and mental health professionals, institutional racism, and mental illness stimatization.^30^ Dermatologists need to be mindful of both structural barriers and biases, ensuring they are well-equipped to assist patients in overcoming these obstacles. Dermatologists should be aware of these difficulties for them to initiate psychiatric treatments while exploring the individual reasons and underscoring the importance of seeking effective treatment.

Furthermore, depression is associated with poor medication adherence, so adequate treatment of depressive symptoms may increase adherence with vitiligo treatment. Treatment of vitiligo may therefore help lower depressive symptoms, and treatment of psychiatric illness may help decrease vitiligo flares or progression. This bidirectional impact of treatment further emphasizes the importance of psychiatric screening and treatment initiation in patients with vitiligo.

Dermatologists play an important role in screening for psychiatric comorbidities in patients with vitiligo and other skin conditions. The American Association of Pediatrics recommends using screening tools such as the Pediatric Symptom Checklist-17 or the Strengths and Difficulties questionnaire for an initial psychosocial assessment of patients.^31^ Use of screening tools or introducing dermatological mental health screening tools may enable early identification, intervention, and prevention of mental health issues associated with vitiligo. However, there is currently no universally accepted screening tool specifically designed to comprehensively evaluate psychiatric comorbidities in vitiligo patients. Fostering collaborative relationships between dermatologists and mental health providers^31^^,^^32^ is crucial for optimizing treatment outcomes, alongside the development and implementation of a standardized screening tool to promptly identify psychiatric conditions and provide tailored care for both dermatological and psychological needs.

Despite the attempts to mitigate potential confounding variables in the study, several limitations exist. First, the retrospective nature of the study hinders the ability to determine a causal relationship between vitiligo and psychiatric comorbidities in pediatric patients with vitiligo, and the relatively small sample size limits the power of our study by affecting the stability of the calculated ORs. Second, diagnostic nuances among providers may exist, particularly for younger patients whose clinical presentation could be attributed to various possible psychiatric diagnoses (ie anxiety versus DBDs). Another limitation of our study is the heterogeneity of the control group, which includes patients with various chronic conditions. While this mirrors the real-world scenario where patients with vitiligo often have other chronic conditions, it may affect the external validity of our comparisons and conclusions. Lastly, the exploration of duration or efficacy of psychiatric treatment in this study was constrained by limited accessibility to documentation.

## Conclusion

Our study demonstrated that vitiligo is linked to a greater prevalence of comorbid psychiatric diagnoses, including depression, DBDs, EDs, GAD, SA, and SI in pediatric African American patients compared to nonvitiligo peers. Therapeutic intervention for mental health concerns remains inadequate in this intersectional population. Pediatric dermatologists have an important role in screening for psychiatric comorbidities, and implementation of appropriate screening tools while treating vitiligo is likely to have a bidirectional positive impact. By better understanding psychiatric comorbidities of African American children with vitiligo, dermatologists can be more aware of pediatric mental health needs and provide appropriate referrals.

## Conflicts of interest

None declared.
